# Early Leukocyte Responses in *Ex-Vivo* Models of Healing and Non-Healing Human *Leishmania (Viannia) panamensis* Infections

**DOI:** 10.3389/fcimb.2021.687607

**Published:** 2021-09-07

**Authors:** Maria Adelaida Gomez, Ashton Trey Belew, Adriana Navas, Mariana Rosales-Chilama, Julieth Murillo, Laura A. L. Dillon, Theresa A. Alexander, Alvaro Martinez-Valencia, Najib M. El-Sayed

**Affiliations:** ^1^Centro Internacional de Entrenamiento e Investigaciones Médicas (CIDEIM), Cali, Colombia; ^2^Universidad IcesiI, Cali, Colombia; ^3^Department of Cell Biology and Molecular Genetics, University of Maryland, College Park, MD, United States; ^4^Center for Bioinformatics and Computational Biology, University of Maryland, College Park, MD, United States; ^5^Pontificia Universidad Javeriana, Cali, Colombia

**Keywords:** *Leishmania Viannia*, PBMCs, leukocytes, host-pathogen interactions, RNA-Seq

## Abstract

Early host-pathogen interactions drive the host response and shape the outcome of natural infections caused by intracellular microorganisms. These interactions involve a number of immune and non-immune cells and tissues, along with an assortment of host and pathogen-derived molecules. Our current knowledge has been predominantly derived from research on the relationships between the pathogens and the invaded host cell(s), limiting our understanding of how microbes elicit and modulate immunological responses at the organismal level. In this study, we explored the early host determinants of healing and non-healing responses in human cutaneous leishmaniasis (CL) caused by *Leishmania (Viannia) panamensis*. We performed a comparative transcriptomic profiling of peripheral blood mononuclear cells from healthy donors (PBMCs, n=3) exposed to promastigotes isolated from patients with chronic (CHR, n=3) or self-healing (SH, n=3) CL, and compared these to human macrophage responses. Transcriptomes of *L. V. panamensis-*infected PBMCs showed enrichment of functional gene categories derived from innate as well as adaptive immune cells signatures, demonstrating that *Leishmania* modulates adaptive immune cell functions as early as after 24h post interaction with PBMCs from previously unexposed healthy individuals. Among differentially expressed PBMC genes, four broad categories were commonly modulated by SH and CHR strains: cell cycle/proliferation/differentiation, metabolism of macromolecules, immune signaling and vesicle trafficking/transport; the first two were predominantly downregulated, and the latter upregulated in SH and CHR as compared to uninfected samples. Type I IFN signaling genes were uniquely up-regulated in PBMCs infected with CHR strains, while genes involved in the immunological synapse were uniquely downregulated in SH infections. Similarly, pro-inflammatory response genes were upregulated in isolated macrophages infected with CHR strains. Our data demonstrate that early responses during *Leishmania* infection extend beyond innate cell and/or phagocytic host cell functions, opening new frontiers in our understanding of the triggers and drivers of human CL.

## Introduction

Clinical manifestations of human infections with *Leishmania Viannia* species (primarily *L. V. braziliensis* and *L.V. panamensis*) range from single self-healing skin lesions, to severe and chronic cutaneous or mucosal disease (Weigle et al., 1993; Murray et al., 2005). Contrary to infections where microorganisms inflict direct damage to the host *via* toxins or cellular injury, symptomatic dermal leishmaniasis (cutaneous, mucosal or muco-cutaneous) results from parasite-elicited immunopathology, and disease severity depends on the degree of immunopathology and concomitant immunoregulation (Murray et al., 2005; Rodriguez-Pinto et al., 2012). For these immunopathologically-driven infections, the early host-microbe interactions and the elicited innate immune responses, together with specific features of the anatomical sites and tissues affected, shape the adaptive responses that contribute to pathology and disease severity. However, the triggers and drivers of immunopathology of dermal leishmaniasis remain largely unknown.

Genotypic and phenotypic variability among *Leishmania* isolates of the same species has been long-recognized. Within the *L. Viannia* subgenus, a number of phenotypically discernable populations (zymodemes) co-circulate in endemic foci of transmission, with *L. V braziliensis* and *L.V. panamensis* being the two species with the highest heterogeneity (Saravia et al., 1998). Distinct host responses as well as intrinsic parasite phenotypes have been correlated with different zymodemes. Disease caused by *L. V. braziliensis* zymodeme 1.1 has been associated with a longer time of evolution in humans (Saravia et al., 1998), and *L. V. panamensis* zymodeme 2.3 has been identified as a population intrinsically resistant to antimony (Fernandez et al., 2014). Thus, parasite factors, as well as their interaction with the host may contribute to the distinct immunological responses unleashed upon infection with different strains of the same *Leishmania* species. Although the specific determinants of such responses are currently unknown, variations in virulence factors involved in modulating the early stages of the host-pathogen interaction [e.g. GP63 copy number or function (Gomez and Olivier, 2010)], could contribute to divergent clinical outcomes of disease.

Characterization of the early *Leishmania-*host interactions has been primarily conducted in the context of isolated phagocyte infections [macrophages, dendritic cells and neutrophils, and predominantly using murine or human cell lines (Cohen-Freue et al., 2007; Ovalle-Bracho et al., 2015)]. We have previously shown that *L. V. panamensis* strains isolated from patients with chronic cutaneous leishmaniasis (CL) induced significantly more expression of pro-inflammatory chemokines and cytokines in human macrophages (Navas et al., 2014), consistent with parasite-elicited hyperactivation of the host-cell inflammatory response driving disease severity. However, during natural vector-borne infections, the initial host-parasite interaction involves a multitude of other immune and non-immune cells, which constitute the cellular micro-environment to which, promastigotes are exposed after inoculation into the human host. Murine models of infection have provided evidence of the early participation of keratinocytes in susceptibility to *L. major* via induction of pro-inflammatory chemokines and cytokines (Ehrchen et al., 2010; Ronet et al., 2019). In humans, keratinocytes were proposed to participate in the development of post-kala azar dermal leishmaniasis, and in the pathologic changes of ulcerating skin lesions (Gasim et al., 1998; Tasew et al., 2010). The participation of non-immune and non-phagocytic immune cells in the early interactions of *L. Viannia* species and the host, and the contribution of those responses to development of symptomatic disease and in disease severity, are not completely understood.

Gene expression profiling of peripheral blood mononuclear cells (PBMCs) responses, both at the basal level and after antigenic recall, has been successfully implemented as a tool to identify immunological gene signatures of clinical and therapeutic outcomes in human infections. Illustrating this, transcriptional profiling of PBMCs has revealed gene signatures associated with immunological protection in human malaria vaccine studies (Moncunill et al., 2020), responsiveness to antiviral treatment in human hepatitis C infections (Orr et al., 2020), and long-lasting immune dysfunction in patients with chronic ebola infection (Wiedemann et al., 2020), among others. More recently, consistency between results from bulk RNA-seq and single cell sequencing has been demonstrated with PBMCs from COVID-19 patients (Arunachalam et al., 2020), supporting the use of bulk transcriptomics to explore immunological signatures of infectious diseases and disease severity.

Antigen-specific recall responses of PBMCs from CL patients has demonstrated that a mixed T_h_1/T_h_2 response, accompanied by immune deregulation, participates in the clinical manifestations and disease severity caused by *L. Viannia* (Carvalho et al., 1985; Saravia et al., 1989; Diaz et al., 2010). In this study, we examined how clinical strains of *L.V. panamensis* which cause different degrees of severity of CL in humans (chronic or self-healing CL), modulate the early responses of PBMCs within the first 24 hours of parasite-host interaction. We hypothesize that these initial PBMC-promastigote interactions contribute to define the nature and magnitude of activated or repressed host functions, ultimately determining the clinical outcome of infection. Identification of immunological signatures associated with development of more severe disease manifestations and triggered during the early phases of infection, will serve as a knowledge base for the development of prognostic tools, ultimately allowing timely intervention for these cases, known to be more refractory to first-line antileishmanial drugs (Amato et al., 2008; Maurer-Cecchini et al., 2009; Bamorovat et al., 2021).

## Materials and Methods

### Isolation of Total WBCs and PBMCs

Peripheral blood samples from healthy volunteers without history of CL and residing in an urban non-endemic city, were collected and processed to obtain total WBCs and PBMCs. WBCs were isolated by centrifugation at 400 g for 15 min at RT and cells were collected from the interface between the plasma and red blood cells. WBCs were incubated in RBC lysis buffer ([150 mM] NH_4_CL, [10 mM] KHCO_3_, [0.1 mM] Na_2_EDTA) for 5 min at RT, washed with PBS, and resuspended in RPMI supplemented with 10% FBS for subsequent procedures. PBMCs were obtained by centrifugation of PBS-diluted (1:1) blood samples over a Ficoll-Hypaque gradient (Sigma-Aldrich) following the manufacturer’s instructions.

### *Leishmania* Strains

Clinical strains were obtained from the CIDEIM BioBank. Strains were originally isolated by needle aspirate of cutaneous lesions or from biopsies of mucosal lesions, propagated in Senekjie´s biphasic blood agar and immediately stored in liquid nitrogen until use. Strains were typed by immunoreactivity to monoclonal antibodies. *L. V. panamensis* strains MHOM/CO/11/5430 (5430chr), MHOM/CO/08/5433 (5433chr) and MHOM/CO/08/5397 (5397chr) were isolated from patients with chronic CL of > 6 months´ evolution. MHOM/CO/87/1320 (1320chr) and MHOM/CO/85/2504 (2504chr) were isolated from lesion biopsies of nasal mucosa from patients with >10 years of muco-cutaneous disease. Strains MHOM/CO/85/2272 (2272sh), MHOM/CO/85/2271 (2271sh), MHOM/CO/89/2189 (2189sh) and MHOM/CO/83/1022 (1022sh) were isolated from CL patients who clinically resolved disease in the absence of any treatment (self-healing CL).

### Infection

Promastigotes were grown at 25°C in Senekjie’s biphasic medium and passed for a maximum of two sub-passages into RPMI 1640 supplemented with 10% heat-inactivated FBS and 5 mg/ml hemin. Ten million WBCs or PBMCs were infected at a 10:1 parasite-to-monocyte ratio with human AB+ serum-opsonized stationary phase promastigotes for 24h. Selection of 24 hours post infection (hpi) as the time to characterize the early interaction of PBMCs and *Leishmania*, was based on prior transcriptomic data showing a strong modulation of gene expression at 4 hpi, and stabilization of these responses at 24 hpi, remaining essentially unaltered up to 72 hpi (Dillon et al., 2015; Fernandes et al., 2016).

### Macrophage Samples

All macrophage samples used were those previously collected and characterized in a previous study from our group (Navas et al., 2014). Briefly, macrophages were differentiated from PBMCs by adherence to cell culture plasticware in serum-free RPMI for 2h, followed by culture for 7 days in RPMI supplemented with 20% FBS at 37°C and 5% CO_2_. Cells were infected for 24h at a 1:10 ratio as described above.

### RNA Isolation and cDNA Synthesis

Total RNA was extracted from uninfected and infected cultured cells using Trizol (Invitrogen, USA) followed by RNA cleanup with RNeasy Mini Kit columns (Qiagen, USA). RNA integrity was assessed using an Agilent 2100 bioanalyzer. For RNA-seq, poly(A)-enriched cDNA libraries were generated using the Illumina TruSeq v2 sample preparation kit (San Diego, CA) and checked for quality and quantity using bioanalyzer and quantitative PCR. For qRT-PCR analyses, RNA was reverse transcribed with RT First Strand Kit (SABiosciences-Qiagen).

### Gene Expression by qRT-PCR

Gene expression analysis of 84 inflammatory genes and receptors was conducted by qRT-PCR using Qiagen PCR Arrays (PAHS-077Z) on a BioRad^®^ CFX-96 detection platform. Gene expression was normalized to a five gene panel composed of β2-microglobulin, hypoxanthine phosphoribosyltransferase-1, β-actin, GAPDH and ribosomal protein L13a. Data was analyzed using the ΔΔCt method and fold change calculated compared to uninfected cells and expressed as 2^-ΔΔCt^. Data was processed and analyzed on the RT² Profiler™ PCR Array Data Analysis online tool provided by the manufacturer.

### RNA-Seq Data Generation, Preprocessing, and Quality Trimming

Paired-end reads (100 bp) were obtained using an Illumina HiSeq 1500. Trimmomatic (Bolger et al., 2014) was used to remove Illumina adapter sequences from reads and to trim bases off the start or the end of a read when the quality score fell below a threshold of 25. Sequence quality metrics were assessed using FastQC (http://www.bioinformatics.babraham.ac.uk/projects/fastqc/).

### Mapping cDNA Fragments, Abundance Estimation, and Data Normalization

Reads were aligned against the human (hg38 revision 91), *L.V. panamensis* (TriTrypDb release 36), and *L.V. braziliensis* (release 26) genomes with TopHat (2.1.0) (Trapnell et al., 2012) using parameters to randomly place multi-matches (-g 1), using an existing set of splice junctions (-G), and using the more sensitive bowtie2 options (–b2-very-sensitive). The resulting accepted hits and mapped reads were sorted and indexed *via* SAMtools (Li et al., 2009) and passed to HTSeq (Anders et al., 2015) for generating count tables.

### Global Data Assessment, Visualization and Differential Expression Analysis

Biological replicates and batch effects were assessed and visualized using the hpgltools (https://github.com/elsayed-lab/hpgltools) R package. The process included creating density plots, boxplots of depth, coefficient of variance, hierarchical clustering analyses based on Pearson’s correlation coefficient and Euclidean distance, variance partition analyses (Hoffman and Schadt, 2016), and principal component analyses before and after normalization. Several combinations of normalization and batch adjustment strategies were evaluated. The normalization methods tested included trimmed median of M-values, relative log expression, and quantile. These were combined with different batch evaluation strategies including: surrogate variable analysis (SVA) (Leek et al., 2012), ComBat (in sva), RUV (Risso et al., 2014), and batch factor removal *via* residuals (performed manually and *via* limma’s (Ritchie et al., 2015) removeBatchEffect function). Normalized data were visualized using log_2_ transformed counts per million reads following filtering to remove low counts (defined as any gene with fewer counts than twice the number of samples or when any sample had fewer than 2 counts).

Differential expression analyses were performed using a single pipeline which performed all pairwise comparisons using the Bioconductor packages: limma, edgeR (Robinson et al., 2010), DESeq2 (Love et al., 2014), EBSeq (Leng et al., 2013), and a statistically uninformed basic analysis. In each case (except EBSeq and the basic analysis), the surrogate variable estimates provided by SVA were used to adjust the statistical model in an attempt to address the batch/surrogate effects. The quality of each contrast was evaluated by the degree of agreement among methods, but the interpretations were primarily informed by the DESeq2 results. Detailed information on the analytical pipeline and scripts is available at (https://github.com/abelew/cideim_early_leukocyte).

Genes with significant changes in abundance (fold change ≥ |1.5| and false discovery rate adjusted *P* values ≤ 0.05) were passed to a few gene set enrichment methods including: GOseq (Young et al., 2010), clusterprofiler (Yu et al., 2012), topGO, GOstats (Falcon and Gentleman, 2007), gProfileR (Reimand et al., 2016) and gene set variation analysis (GSVA) (Hanzelmann et al., 2013). Gene ontology analyses were supplemented with manual data curation. GSVA was performed to produce an enrichment score for each gene set per sample. These scores were passed to limma to evaluate the difference in GSVA score distributions for each gene set in the samples. Limma results were then filtered according to log2 fold change, adjusted p-value, and maximum GSVA score mean. GSVA was conducted using the immunologic signature gene sets publicly available at GSEA|MSigDB (C7 collection) (Liberzon et al., 2011). Network analyses were done with STRING 11.0 (Szklarczyk et al., 2019).

### Detection of *Leishmania* RNA Virus (LRV)

Total RNA was extracted from stationary-phase promastigotes using TRIzol (Invitrogen, USA), and cDNA was synthesized using a high-capacity cDNA reverse transcription kit (Applied Biosystems). LRV was detected by qRT-PCR using the primer sets described by (Zangger et al., 2013): Forward 5′-CTG ACT GGA CGG GGG GTA AT-3′ and Reverse 5′-CAA AAC ACT CCC TTA CGC-3′, derived from LRV1-4 genome sequences (GenBank accession number: NC_003601). As a quality control procedure for nucleic acids, a 372 bp fragment of the *Leishmania* β-tubulin gene was also amplified. Positive and negative controls for LRV were included in each run: *L. V. guyanensis* M5313 (WHI/BR/78/M5313 – LRV+) and *L. V. panamensis* (MHOM/CO/2002/3594 stably transfected with the luciferase reporter gene, L.p.LUC 001, LRV-).

## Results and Discussion

### Profiling of Inflammatory Mediators in WBCs and PBMCs Reveals a Similar Signature

**Figure 1:**
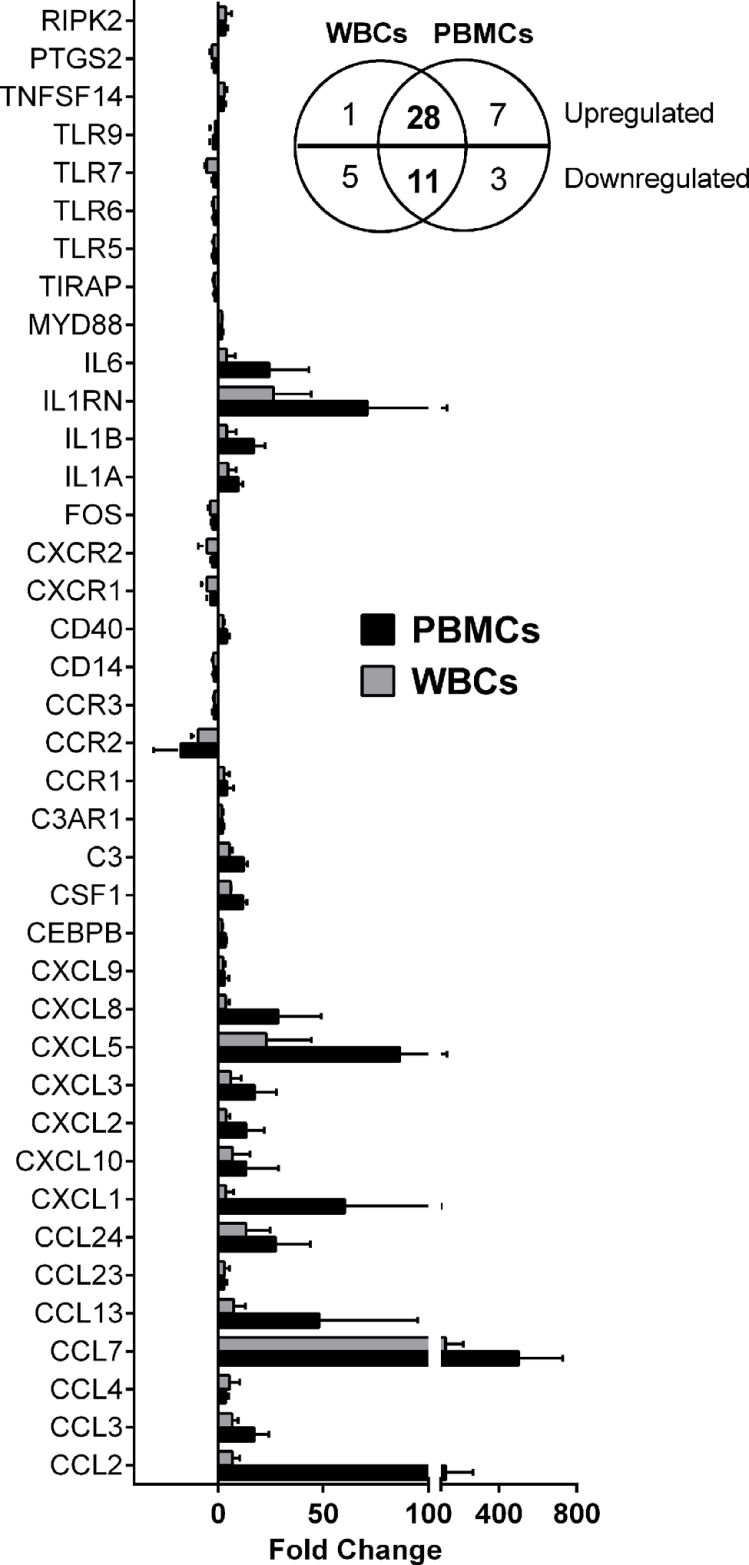
Differentially expressed genes in WBCs and PBMCs upon *L. V. panamensis* infection. Fold change gene expression (infected *vs.* uninfected) of genes modulated in WBCs and PBMCs from three healthy donors after *ex vivo* infection with *L. V. panamensis* for 24h. Insert: Venn diagram representing the number of upregulated and downregulated genes.

The macrophage-*Leishmania* infection model has been consistently used as the preferred *in vitro* and *ex vivo* experimental system, because it portrays the interaction of the parasite and its primary host cell. However, recruitment of other cells to the inflammatory microenvironment generated during the vector blood meal, implies that the early interaction between the parasite and the host extends beyond the macrophage-*Leishmania* contact. Different experimental approaches and cell preparations can be used to explore these *Leishmania*-leukocyte interactions (e.g. whole blood, total white blood cells -buffy coat-, isolated PBMCs). To select the *ex vivo* cellular system for our experiments, we performed a comparative expression profiling of 84 immune response genes in total human white blood cells (WBCs) and PBMCs collected from healthy donors (n=3), which were then exposed to *L.V. panamensis* promastigotes for 24 hours. Eleven genes (*ccl11, ccl16, ccl21, ccl8, crp, IL17a, il23r, il9, kng1, nos2* and *sele*) were not detected in either PBMCs or WBCs. *Il22* was only detected in infected PBMCs from one donor, and *ccl19* was not detected in WBCs, but was induced in infected PBMCs (**Table S1**). 56 genes were modulated by exposure to *L. V. panamensis* (fold change, FC ≥ |1.5|), and 39 were common in PBMCs and WBCs (28 upregulated and 11 downregulated). Modulation of *il1r, il1rap* and *tlr1* was mainly found in WBCs, which is consistent with the predominant expression in granulocytes (Uhlen et al., 2015). The magnitude of modulation was consistently higher in PBMCs than WBCs, in line with higher transcriptional activity of mononuclear compared to polymorphonuclear cells (Ericson et al., 2014; Grassi et al., 2018) **(****Figure 1****)**. The similarity of gene expression profiles supports the use of PBMCs as an informative cell population to explore the predominant immunological signatures elicited during the early stages of *L.V. panamensis* infections.

### Exposure of PBMCs to *L. Viannia* Modulates Transcriptional Signatures of Lymphocyte Activation

PBMCs from three healthy donors from a non-endemic area, were exposed for 24h to stationary phase promastigotes isolated from patients with chronic (CHR, n=3) or self-healing (SH, n=3) CL. *Leishmania* virulence factors such as the major surface metalloprotease GP63, lipophosphoglycan (LPG), and the *Leishmania* RNA virus (LRV), contribute to disease severity in murine models of infection (Beverley and Turco, 1998; Gomez et al., 2009; Ives et al., 2011). However, a clear relationship of these and other virulence determinants in the clinical outcome of human infections remains elusive. We evaluated the presence of LRV in all clinical strains used in this study. All strains except for the positive control *L. V. guyanensis* M5313, which was LRV(+) with an avergae Ct value of 24,3.

Poly(A)-enriched cDNA libraries were constructed and 100 bp paired-end reads were generated (**Table S2** and **Figure S1**). Each sample from infected cells consisted of a pool of mixed RNAs from parasites and human macrophages. Post-experimental authentication of *Leishmania* strains used in these experiments was carried out by SNP profiling of parasite-derived sequences. This confirmed that all strains used corresponded to *L.V. panamensis*, except for strain MHOM/CO/11/5430 which was identified as a mixed *L. V. panamensis/L. V. braziliensis* isolate.

**Figure 2:**
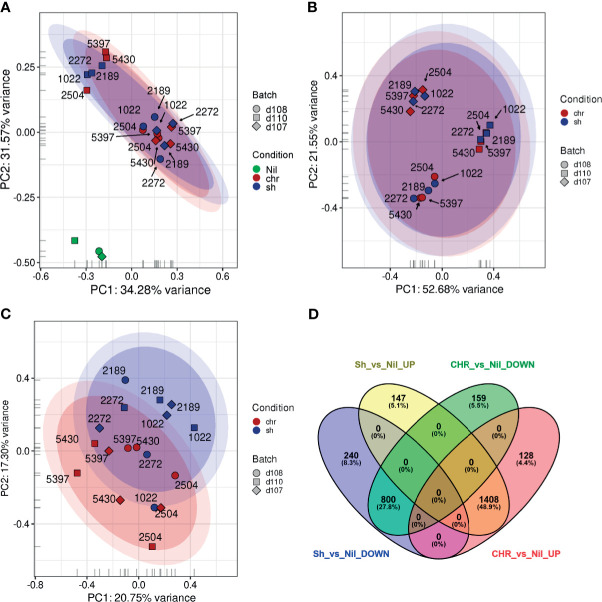
Principal Component Analysis plots and Differential Expression analyses of PBMCs exposed to SH and CHR strains. An initial PCA was performed **(A)** using log_2_, quantile, count per million (cpm), low-count-filtered data and displayed a strong clustering with respect to the three donors (represented as the different symbol shapes). **(B)** This donor-specific effect was made even clearer when the uninfected samples (green) were removed from the analysis. **(C)** When surrogate variable estimates from SVA were included, it became possible to visualize differences between the PBMCs exposed to CHR (red) and self-healing (blue) strains. **(D)** Venn diagrams showing the relationships between the overlaps of differentially expressed genes in SH *vs.* uninfected (Nil) and CHR *vs.* uninfected samples.

A principal component analysis (PCA) showed that a significant amount of the variance observed in the host transcriptomic data resulted from either the infection status (infected *vs.* uninfected, **Figure 2A**) or, in the absence of uninfected samples, from inter-donor variability (**Figure 2B**). To account for these sources of variance, an adjusted statistical model was developed using SVA. A PCA of the SVA-adjusted data showed a partial separation of samples based on infection with CHR *vs.* SH strains (**Figure 2C**).

Differential expression (DE) analysis of the transcriptomes from PBMCs exposed to SH strains against CHR strains yielded no statistically significant DE genes. Considering that most of the variance between samples was attributed to either donor variability or infection status, we conducted three additional analyses: 1) an independent DE analysis of infection with CHR *vs.* SH strains for each donor; 2) a grouped analysis of PBMCs from the three donors infected with SH strains against uninfected controls, and 3) a grouped analysis of PBMCs from the three donors infected with CHR strains against uninfected controls. For the independent donor analyses, transcriptomes of PBMCs from one donor did not identify statistically significant DE genes. For the second and third donors, only 3 and 28 DE genes were found respectively (**Table S3**). Grouped analysis of infected PBMCs against uninfected controls yielded over 2800 significantly DE genes in infections with either SH or CHR strains. Of these, 2208 (78.8%) were common to CHR and SH infections: 1408 upregulated and 800 downregulated (cut off values: p ≤ 0.05; log_2_FC ≥ |0.58|, i.e. fold change ≥ |1.5|), **Figure 2D**. Despite the statistical significance of this enrichment analysis, the biological information extracted from these GO categories is limited.

Among up- and down-regulated genes, the top 10 significantly enriched categories (ranked by % representation within the dataset) corresponded to broad categories related to immune functions, cell signaling, transcription, and cell proliferation, among others (**Table S4**).

**Figure 3:**
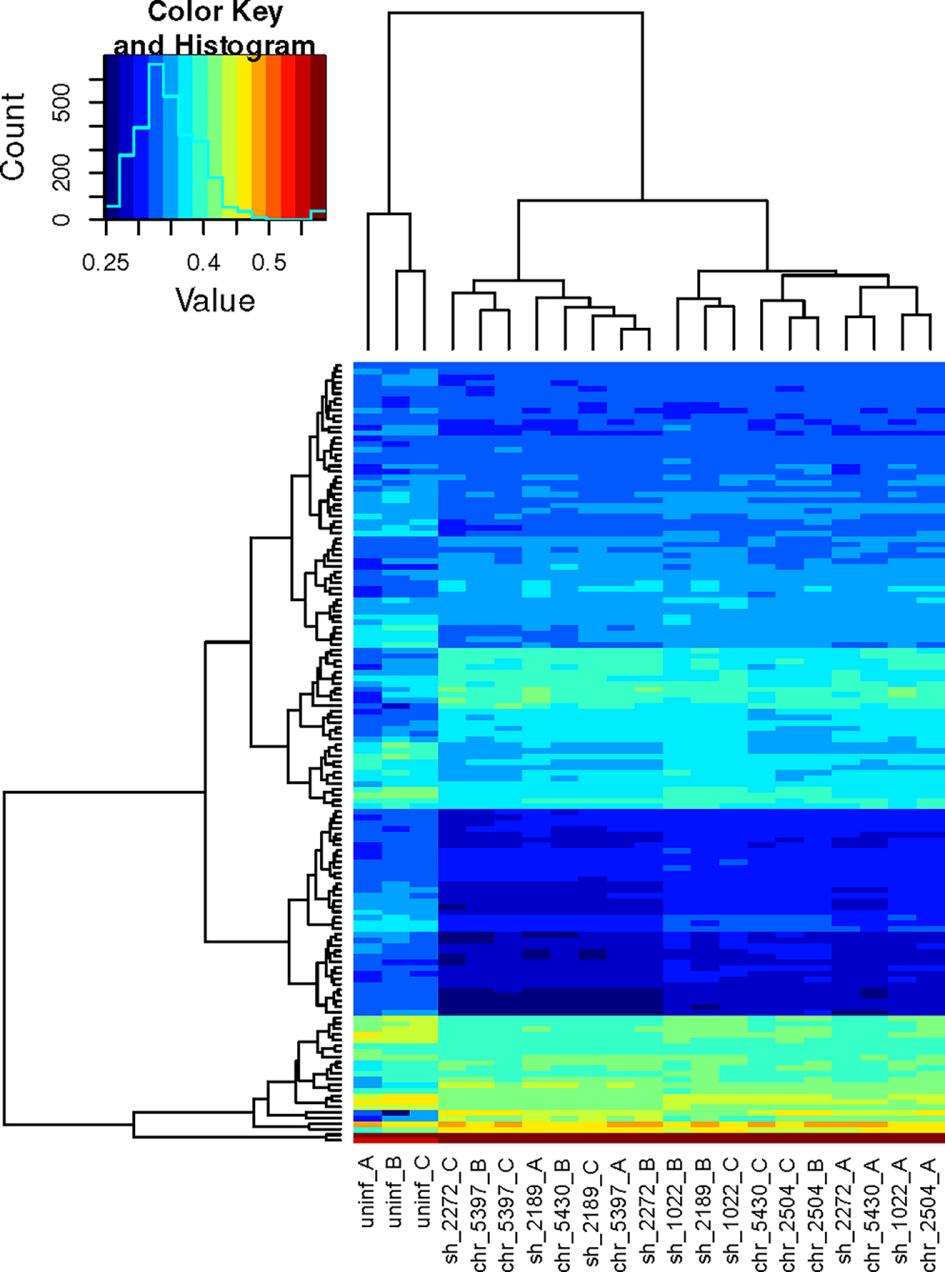
Hierarchical clustering heatmap of significant mSigDB c7 gene sets. GSVA was performed using the normalized expression data and the mSigDB c7 gene sets. The resulting scored gene sets were examined using limma and by *de-novo* comparisons of the mean group scores in order to extract the most significant gene sets. The remaining scores were passed to a hierarchical clustering algorithm and plotted. Each column corresponds to an independent sample, each row to a unique gene set. Columns 1- 3, uninfected PBMCs from three independent healthy donors (A–C). Columns 4-18, infected PBMCs. Sample codes correspond to the phenotype of the disease caused by the *Leishmania* strain (sh, self healing; chr, chronic), followed by the strain code and the PBMC donor.

To provide a more insightful biological interpretation of the effect of early exposure of PBMCs to *L.V. panamensis*, and to explore the potential contribution of specific cell types to the bulk transcriptomic profile of PBMCs, we conducted gene set variation analysis (GSVA) using the immunologic signature gene sets publicly available at GSEA|MSigDB (C7 collection) (Liberzon et al., 2011). 140 categories were significantly and differentially enriched in *L.V. panamensis* infected *vs.* uninfected PBMCs (**Figure 3** and **Table S5**), independent of whether cells were exposed to CHR or SH strains. Interestingly, 37 gene sets were derived from experiments involving microbial agents, including infections with old world *Leishmania* species (*L. donovani and L. major*) and other intracellular microbes (*Toxoplasma gondii* and *Mycobacterium tuberculosis*). This potentially shows common early response mechanisms against intracellular pathogens.

Among enriched categories, 47 corresponded exclusively to innate cell signatures (monocytes, macrophages, NK cells, dendritic cells), while 56 were related to signatures derived from adaptive immune cells (CD8 and CD4 T cells, Th17, Tregs, T follicular cells, B cells) **(****Table S5****)**. This indicates that as early as 24h after interaction, *Leishmania* modulates adaptive immune cell functions in PBMCs from previously unexposed healthy individuals, revealing a largely unexplored and exploitable dimension of the host-pathogen interactome in Leishmania infections. The mechanism(s) mediating this rapid activation, especially of naïve T cells, remains to be determined, but could occur *via* bystander (antigen-independent) activation of naïve or memory T cells (Kim and Shin, 2019; Lee et al., 2020), cross-reactivity of memory T cells, or potentially *via* antigen-presentation by circulating fibrocytes (Chesney et al., 1997; Grab et al., 2004). Interestingly, bystander activation of *Lysteria*-specific and *LCMV-*specific memory T-cells has been reported in chronic *L. donovani* infection of *Lysteria-*immune mice (Polley et al., 2005), and in acutely *L. major* infected mice, respectively (Crosby et al., 2014).

### Exposure of PBMCs to SH and CHR Strains Differentially Modulate Genes Related to IFN Responses and the T Cell Synapse

We next analyzed the gene set differentially modulated by SH and CHR infections. A total of 387 genes were uniquely modulated in PBMCs after exposure to SH strains (240 down- and 157 up-regulated), and 287 were unique to infection with CHR strains (159 down- and 128 upregulated) **(****Table S6****)**. Functional interpretation of DE genes was performed *via* GO enrichment, complemented by network analyses and manual curation. For infections with SH strains, no significantly enriched GO categories were found. For infections with CHR strains, a single GO category was enriched (FDR=0.00029): Type I IFN signaling (reactome pathway HSA-1606322) (**Table S7**).

Upon manual curation, 17 functional categories were defined (all containing ≥ 5 genes, except for “antimicrobial activity”). Four categories (metabolism of macromolecules, cell cycle/proliferation/differentiation, immune signaling and vesicle trafficking/transport) were common to infections with SH and CHR strains (**Table S7**). Genes involved in metabolism of macromolecules (lipid, glycan and nucleic acids metabolism) were found in both upregulated and downregulated gene lists, however, most of these genes were significantly downregulated in infected PBMCs. Rapid downregulation (at 4hpi) of fatty acid metabolism, amino acid catabolism and glycan degradation has been previously documented in murine and human macrophages infected with *L. major* (Dillon et al., 2015; Fernandes et al., 2016); here we show that this is maintained as long as 24hpi. This suggests that basic metabolic functions are stalled during the early time-points of *Leishmania* infection in both phagocytic host cells and other blood leukocytes, potentially revealing an early re-programming of basic metabolic functions towards a state of immune cell activation (O'Neill et al., 2016).

Downregulation of central elements of cell cycle progression and pro-growth signaling (MAPK and PI3K), and concomitant induction of genes involved in oxidative phosphorylation and intracellular vesicle trafficking, were also observed (**Table S7**). Downregulation of genes involved in cell cycle and metabolism of macromolecules, and induction of genes of the tricarboxylic acid (TCA) cycle, is consistent with a state of arrested cell cycle progression. High metabolic demands (provided by metabolism of macromolecules, and not the TCA cycle) are required during cell proliferation and differentiation, while oxidative phosphorylation is a major metabolic pathway in non-proliferative cells (O'Neill et al., 2016). These data further support that the early interaction of *L. Viannia* with PBMCs leads to reduced metabolic capacity, potentially skewing immune cells towards mechanisms of fast energy production to support rapid immune cell activation in otherwise metabolically “paused” cells (Alonso and Nungester, 1956; Newsholme et al., 1986).

Among the uniquely downregulated genes in PBMCs infected with SH strains were primary cilium proteins (**Table S7**). The primary cilium was long considered a vestigial organelle (Cassioli and Baldari, 2019), and re-discovered as a signaling hub (Nachury and Mick, 2019). Although hematopoietic cells do not have primary cilia, a more recent understanding of the T cell immunological synapse (IS) suggests participation of ciliary proteins in IS formation (Cassioli and Baldari, 2019). The assembly and function of the IS and the primary cilium depend on cytoskeleton dynamics and polarized vesicle trafficking, and evidence of strong modulation of these processes was found in the transcriptomic profiles of *L. Viannia*-infected PBMCs (**Table S7**). Within the first hour of interaction between T-cells and antigen presenting cells (APC), an actin-rich invasive pseudopodia emerges from T-cells and probes deep within the APC. The actin cap is then cleared to allow orientation of the microtubule organization center (MTOC) to the IS, resulting in a matured IS (Ueda et al., 2015). Interestingly, disruption of the MTOC does not impact the level of cytokine production in T cells; however, it does change the “directionality” of cytokine secretion so that it is no longer directed at the IS (Ueda et al., 2015). Dampened expression of molecules involved in primary cilia/IS formation during infection of PBMCs with SH strains suggests a cytokine micro-environment that may lead to subtler activation of APCs during interaction with T cells, potentially limiting excessive pro-inflammatory cytokine production that can result in uncontrolled inflammation and immunopathology (Scott and Novais, 2016).

### Macrophage Functions Are Central Drivers of the Divergent Immune Responses Elicited Upon SH and CHR Infections

**Figure 4:**
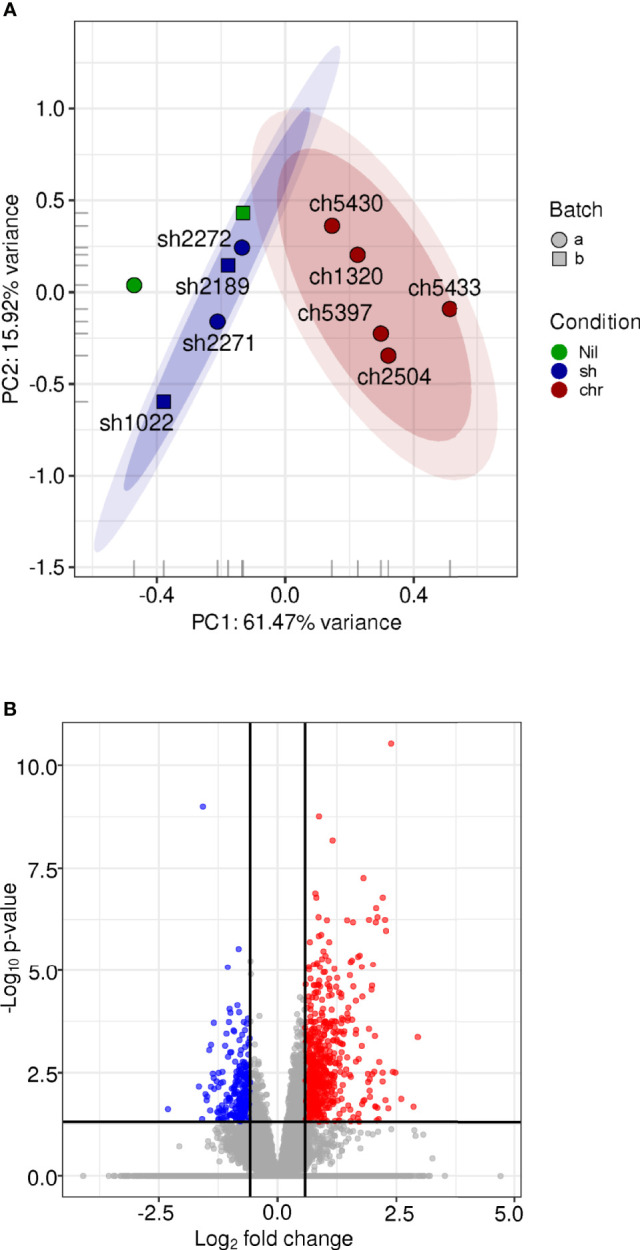
Gene expression profiles of human macrophages infected with SH and CHR L. V. panamensis strains CL. The *(H)* sapiens RNA-seq expression data for annotated coding genes after low-count filtering (7,019 genes remaining) and log_2_[quantile(cpm)] normalization was examined *via* PCA **(A)**. Each glyph represents an experimental sample where the color represents the infection state, the shape represents the experimental batch. The 0.90, 0.95 confidence intervals are shown as shaded ovals. Differential expression analysis was performed using DESeq2, including experimental date (batch) in the statistical model. The volcano plot **(B)** shows the distribution of significance (-log adjusted *P* value) with respect to log_2_-fold change. Points colored in blue are deemed significantly downregulated in the macrophage samples infected with CHR *vs.* SH strains while those in red are significantly up-regulated.

We explored the effect of SH and CHR infections in macrophages as one of the central APCs and principal host cells for *Leishmania*. Total transcriptomic profiles were obtained from primary macrophages infected for 24 h with SH and CHR strains [RNA samples used here were those previously reported in (Navas et al., 2014)]. PCA showed clustering of samples by disease phenotype (**Figures 4A** and **S2**). Macrophages infected with SH strains clustered together and close to uninfected cells, indicating minimal modulation of macrophage gene expression by SH strains, and supporting the controlled APC response that also emerged from SH-infected PBMC transcriptomes.

**Figure 5:**
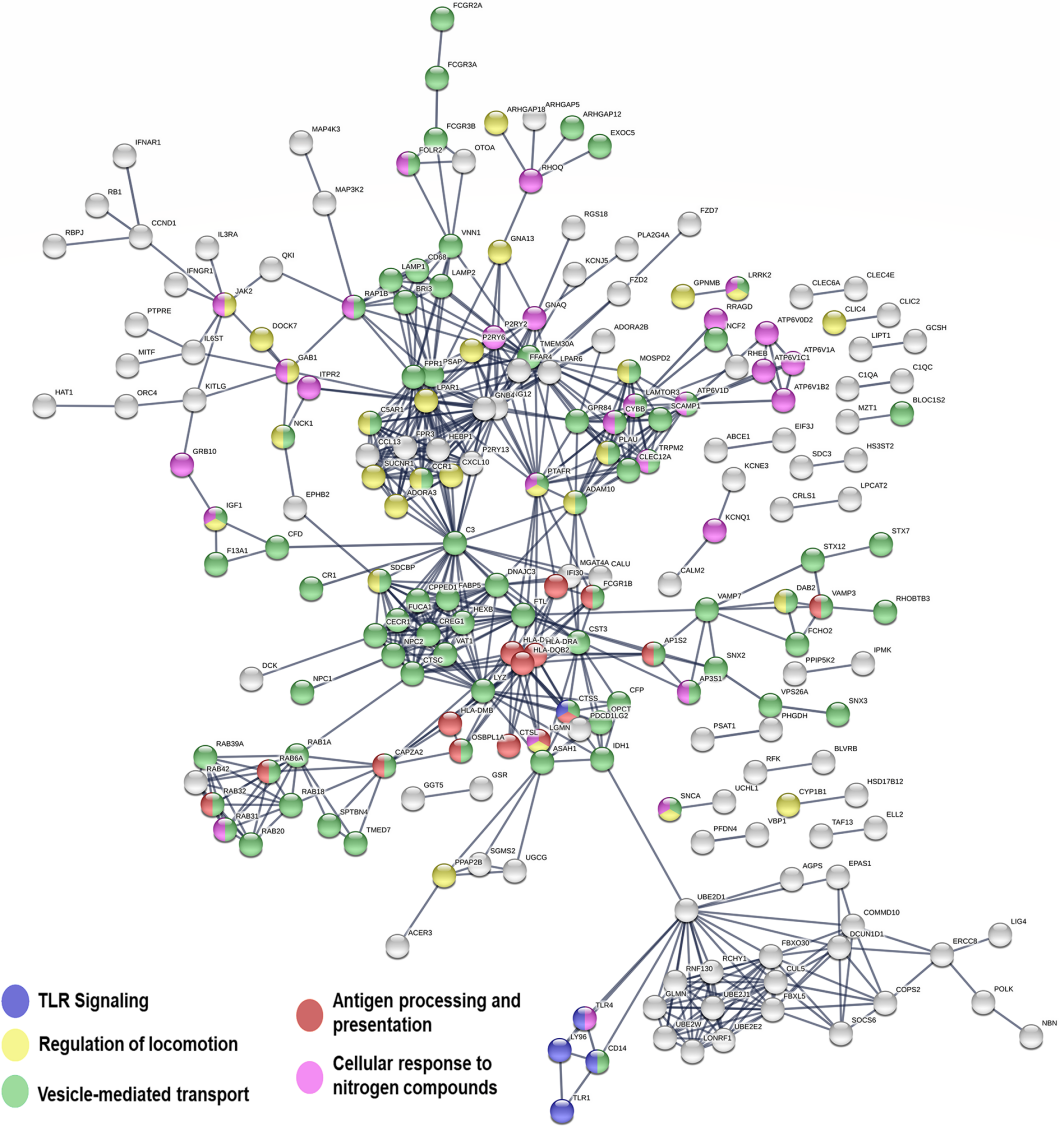
Network representation of genes significantly upregulated in macrophages infected with CHR *vs.* SH strains. Line thickness represents the strength of data support (displaying relationships > 0.9 confidence value). Interaction sources for network building in STRING included experiments, databases, co-expression, neighborhood, gene fusion and co-occurrence. Nodes not in networks are hidden.

Differential gene expression analysis of macrophages infected with CHR *vs.* SH strains identified 884 DE genes (**Figure 4B** and **Table S8**), representing 30% more DE genes compared to those in PBMCs. The macrophage DE gene profile consisted predominantly of upregulated inflammatory response genes in infections with CHR *vs.* SH strains [also previously reported in microarray and qRT-PCR datasets of *L.V. panamensis* infected macrophages (Ramirez et al., 2012; Navas et al., 2014)]. In infections with CHR strains, up-regulation of chemokines involved in monocyte (CCL2, CCL13 and CCR1) and CD4^+^-T_H_1 (CXCL10) activation and recruitment (**Figure 5** and **Table S8**), as well as genes associated with TLR and IFN signaling (TLR4, CD14, TLR1, IFNGR1, IFNAR1, JAK2) were found. In addition, an upregulation of complement components including C3, C1QA, C1QC, CFD and CFP (complement factors D and properdin), ficolin (FCN1), CR1 and the anaphylatoxin receptor C5AR1 (and downstream signaling molecules Rap1b, Rap2a) was observed, which have been associated with a potent induction of inflammation and cell recruitment during acute and chronic inflammatory processes.

Genes involved in opsonophagocytosis and vesicle mediated transport were also induced in CHR-infected macrophages relative to SH-infected cells (**Figure 5** and **Table S8**). Among those were CR1, properdin and C3b, which have been associated with opsonophagocytosis of intracellular pathogens (Rosales and Uribe-Querol, 2017; Lu et al., 2018). Complement receptors participate in uptake of *Leishmania* by macrophages, and a direct relationship between permissiveness of macrophage infection, CR1 and CR3 activity, and chronic CL caused by *L. V. panamensis* has been reported (Robledo et al., 1994). Increased C3 gene expression has been demonstrated in lesion biopsies from CL patients unresponsive to antimonial treatment, potentially leading to enhanced recruitment of polymorphonuclear cells to the affected tissues, promoting immunopathology and a non-healing phenotype (Navas et al., 2020). Induction of multiple Fc gamma receptors (FcgR, including the high affinity receptor FcgR1 as well as low affinity receptors 2a, 2b, 3a and 3b), and a number of genes involved in vesicle-mediated transport (sortins, syntaxins), phagolysosomal acidification (Rab39A, Rab32, Rab20, Vamp7, lamp1, lamp2, V-ATPase subunits among others), and antigen processing and presentation (MHC-II molecules, lyzozyme, cathepsine L) was also observed in macrophages infected with CHR strains, supporting enhanced opsonophagocytosis (Lu et al., 2018) and intracellular parasite killing. Consistent with this hypothesis was the finding that parasite loads were lower in primary human macrophages infected with CHR compared to SH strains (**Table S2**, **Figure S3**).

## Concluding Remarks

The exploration of transcriptomic signatures from both isolated human primary macrophages and PBMCs consistently provides evidence that pathology and severity of CL infection is determined, at least in part, by the immune response. Chronic CL is characterized by the induction of pro-inflammatory leukocyte responses, while benign, self-resolving CL, may be primarily marked by limited and controlled T-cell and APC activation. The early interaction of PBMCs and *L. Viannia* provides a more comprehensive view of additional mechanisms involved in the establishment of infection in the human host. Downregulation of genes involved in biosynthesis and metabolism of macromolecules, cell cycle progression and cellular proliferation, together with upregulation of functional categories related to glycolysis and oxidative phosphorylation, suggest that the parasite-host interplay of *Leishmania* and PBMCs drives cellular functions and energy production towards immunological activation rather than to promote metabolism of macromolecules. Whether this immunological activity is skewed towards a pro-inflammatory environment or a controlled adaptive response, emerges as a potential key aspect for defining the course of human CL. Results from this study instigate further explorations of the *Leishmania*-host interactions involving functional aspects of B and T lymphocytes in the early response to infection (within the first 24 hours of contact). Whether the functions modulated in these cells are dependent on APCs, are a direct effect elicited by the parasite, or are a bystander mechanism of adaptive cell activation, remains to be determined.

## Data Availability Statement

All sequence data is publicly available in NCBI’s Short Read Archive (SRA) under bioproject PRJNA685835 (https://www.ncbi.nlm.nih.gov/bioproject/?term=PRJNA685835).

## Ethics Statement

This study was approved and monitored by the institutional review board for ethical conduct of research involving human subjects of the Centro Internacional de Entrenamiento e Investigaciones Médicas (CIDEIM) in accordance with national (Resolution 008430, República de Colombia, Ministry of Health, 1993) and international (Declaration of Helsinki and amendments, World Medical Association, Fortaleza, Brazil, October 2013) guidelines. All individuals voluntarily participated in the study and written informed consent was obtained from each participant.

## Author Contributions

MG, MR-C, AN, and NE-S contributed to conception and design of the study. AM-V conducted patient recruitment and all clinical management of study participants. AN, MR-C, JM, AB, and LD performed the experiments. AB, MG, and NE-S performed the statistical analysis. MG wrote the first draft of the manuscript. AB, AN, JM, LD, TA, and NE-S wrote sections of the manuscript. All authors contributed to the article and approved the submitted version.

## Funding

This research was funded in part by the American Society of Tropical Medicine and Hygiene Gorgas Memorial Institute Research Award, and received support from NIAID/NIH U19AI129910 and Wellcome Trust 107595/Z/15/Z.

## Author Disclaimer

The content is solely the responsibility of the authors and does not necessarily represent the official views of the National Institutes of Health or other agencies.

## Conflict of Interest

The authors declare that the research was conducted in the absence of any commercial or financial relationships that could be construed as a potential conflict of interest

## Publisher’s Note

All claims expressed in this article are solely those of the authors and do not necessarily represent those of their affiliated organizations, or those of the publisher, the editors and the reviewers. Any product that may be evaluated in this article, or claim that may be made by its manufacturer, is not guaranteed or endorsed by the publisher.
