# Supplementary material for: Early Leukocyte Responses in Ex-Vivo Models of Healing and Non-Healing Human Leishmania (Viannia) panamensis Infections
Source: Front Cell Infect Microbiol. 2021 Sep 7;11:687607. doi: 10.3389/fcimb.2021.687607 (PMC8453012; doi:10.3389/fcimb.2021.687607)
Supplement: Supplementary file 1 [file DataSheet_1.docx]

**Supplementary Materials**

**Early leukocyte responses in *ex-vivo* models of healing and non-healing human *Leishmania (Viannia)* infections**

Maria Adelaida Gomez^1,2†^, Ashton Trey Belew^3,4^, Adriana Navas^1^, Mariana Rosales-Chilama^1,2^, Julieth Murillo^1,5^, Laura A.L. Dillon^3,4^, Theresa A. Alexander^3^, Alvaro Martinez-Valencia^1^, Najib M. El-Sayed^3,4†^

^1^Centro Internacional de Entrenamiento e Investigaciones Médicas (CIDEIM), Cali, Colombia

^2^Universidad ICESI, Cali, Colombia

^3^Department of Cell Biology and Molecular Genetics, University of Maryland, College Park, Maryland, USA

^4^Center for Bioinformatics and Computational Biology, University of Maryland, College Park, Maryland, USA

^5^ Pontificia Universidad Javeriana, Cali, Colombia

**Supplementary Materials and Methods**

**Sequence Filtering and Alignments**

**Scripts used.** Italicized directory names (ending in /) refer to directories within the repository for this project, italicized scripts are provided in the *scripts/* directory, and italicized data files reside in the *data/* directory. Most preprocessing tasks performed were generated via the *cyoa* command line utility, available at: <http://github.com/elsayed-lab/CYOA>. Most later processing tasks were performed with the aid of the R package *hpgltools*, available at: <http://github.com/elsayed-lab/hpgltools>.

**Annotation Collection.** Parasite genomes and annotation information were downloaded from the TriTrypDb revision 26, available at: <http://tritrypdb.org/common/downloads/release-26>. The human genome and annotations revision GRCh38.91 were taken from biomart[(Griffith and Griffith 2004)](https://paperpile.com/c/mMFMCJ/K018), and accessed via the biomaRt[(1)](https://paperpile.com/c/xCaUgR/VhY4) api. Other derived databases (gene ontology/KEGG/etc) were either taken directly from biomart or aggregated from the tritrypdb into the AnnotationDbi interface.

Supplementary Figure Legends

**Figure S1. Diagnostics used to globally assess the data and identify likely confounding effects.** RNA-seq was performed on an Illumina HiSeq-1500 using human peripheral blood monocyte-derived macrophages infected with strains which cause either a chronic or self-healing infection. (**A**) Sample coverage. A potentially strong batch effect associated with coverage was noted when plotting the number of reads collected from each sample. (**B**) Sample clustering before including experimental batch (RNA isolation date) in the statistical model. Normalized expression values were used to generate a Euclidean distance heatmap. This made evident the potential confounding effect of coverage/experiment date based on the observed clusters.

**Figure S2. Raw data metrics of transcriptomic profiles of primary macrophages infected with CHR and SH strains** (**A**) Library sizes including all feature types (e.g. CDS, non-coding RNAs, splice variants, etc.), (**B**) Distance heatmap generated from the raw counts data including all features calculated by Euclidean pairwise distances between samples of human primary macrophage infected with strains from patients with chronic CL (chr) and from patients with self-healing disease (sh), (**C**) Principal component analysis plot of raw counts data.

**Figure S3**. **Parasite load in primary human macrophages.** Time-course experiment (8h, 24h and 48h) of *L. V. panamensis* infection of primary human macrophages from three healthy donors. One representative CHR strain (L.p. 2504) and one SH strain (L.p. 2272) were used for these experiments. Parasite load was determined by qRT-PCR using the *Leishmania* 7SLRNA transcript as target. For quantification of human cells, the Tata Box Binding Protein (TBP) transcript was quantified. Parasite loads are presented as the number of parasites normalized to the number of macrophages in the sample. Error bars represent the standard error of the mean.

**Table S1.**  **Gene expression of inflammatory mediators in PBMCs and WBCs**

**Table S2. Experimental Design.** Samples are listed (Column A) using an internal lab sample identifier (HPGL----, Column B), which is referenced in the records stored at the Short Read Archive (Column C). Four donors (Column E) participated in these experiments. The sample type (Column D) was either peripheral blood mononuclear cells or macrophages. The infection status (E) was either infected or uninfected. Strain used were included in column G. The macrophage experiment was performed (H) in two batches (a or b) and the PBMC experiment was a single batch. The final columns describe the reads observed after (I) trimming with trimmomatic, mapping the reads (J) against the human genome (Hg38-91), the *Leishmania panamensis* (L) and the *Leishmania braziliensis* (N) genomes from the TriTrypDB version 26. The percentage reads mapped was recorded for the human genome (K) and parasite (M, O).

**Table S3. Differential gene expression analyses for each of the three donors.** Pairwise differential expression analyses were performed using DESeq2, the contrasts for analysis were infection with SH vs. CHR strains for each independent donor. The row names (A) were set to the ensembl gene ID. To aid in recognizing genes of interesting function, the HGNC symbol was recorded (C) along with each gene’s description (D). Following the annotation data, statistics from DESeq2 (F,G): the log2 FC, adjusted p-value.

**Table S4: Gene categories derived from the database for annotation, visualization and integrated discovery (DAVID) v6.8**

**Table S5: Gene Sets identified by GSVA and deemed significant when comparing the uninfected vs. all infected PBMC samples.** The expression data was passed to GSVA, the resulting scores and gene set annotations are provided in the first worksheet. Each row name is a mSigDB category (Column A), followed by the reference links (Column B), Entrez gene IDs in each category (Column C), a brief description of each category (Column E), the authors of each study (Column F), the PubMed ID of the associated study (Column G), a manually curated classifier of adaptive vs. innate (Column H), and the scores for each sample. The second worksheet contains all scores from GSVA. The third worksheet contains a likelihood score for each category acquired by examining the distribution of the mean scores by category.

**Table S6: Differential Expression Analyses of PBMCs.** Contrasts were performed by independently comparing the CHR and SH samples vs. the uninfected (Nil) samples. The first two worksheets comprise these results with the same columns as Table S3. The third worksheet combines these for genes which have a log_2_FC ≥ |0.58| (1.5 fold change) and was used to define the set of genes shared in both contrasts (SH vs. Nil and CHR vs. Nil). The fourth sheet contains the remaining unique genes in each contrast.

**Table S7. Manually curated functional gene categories modulated in PBMCs upon infection with SH and CHR strains**

**Table S8. Differential Expression Analyses of Primary Macrophages.** Pairwise differential expression analyses were performed using DESeq2. The primary contrast used in our downstream analyses is chronic vs. self-healing (sheet chr_vs_sh). Columns are as follows: The row names (A) were set to the Ensembl gene ID and transcript version (B) followed and each gene’s description (C). Included are statistics from DESeq2 (E-J): the log_2_FC, adjusted *P*-value, mean values, log FC standard error, t statistic, and *P*-value.
