# Supplementary figures and images for: Early Leukocyte Responses in Ex-Vivo Models of Healing and Non-Healing Human Leishmania (Viannia) panamensis Infections
Source: Front Cell Infect Microbiol. 2021 Sep 7;11:687607. doi: 10.3389/fcimb.2021.687607 (PMC8453012; doi:10.3389/fcimb.2021.687607)

Figure S1

A

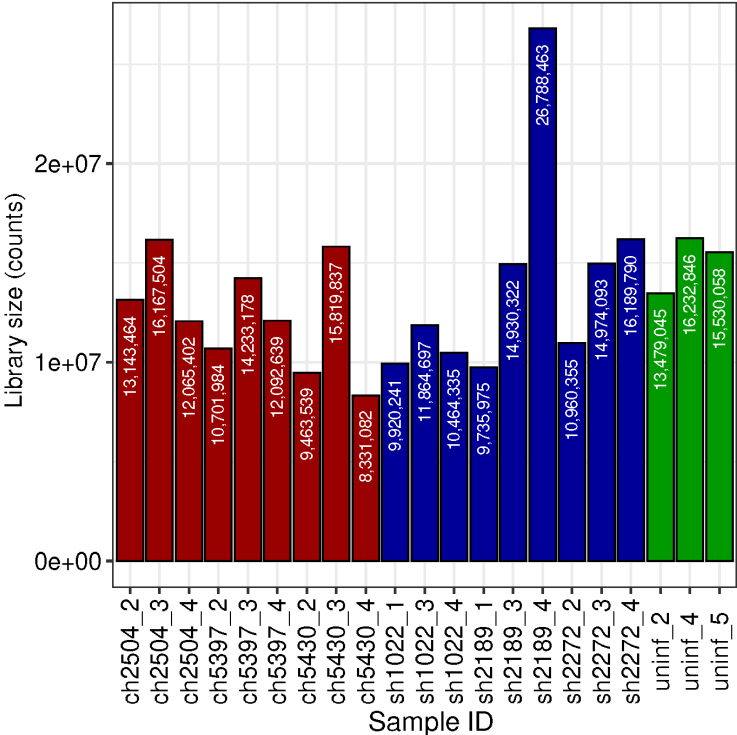

B

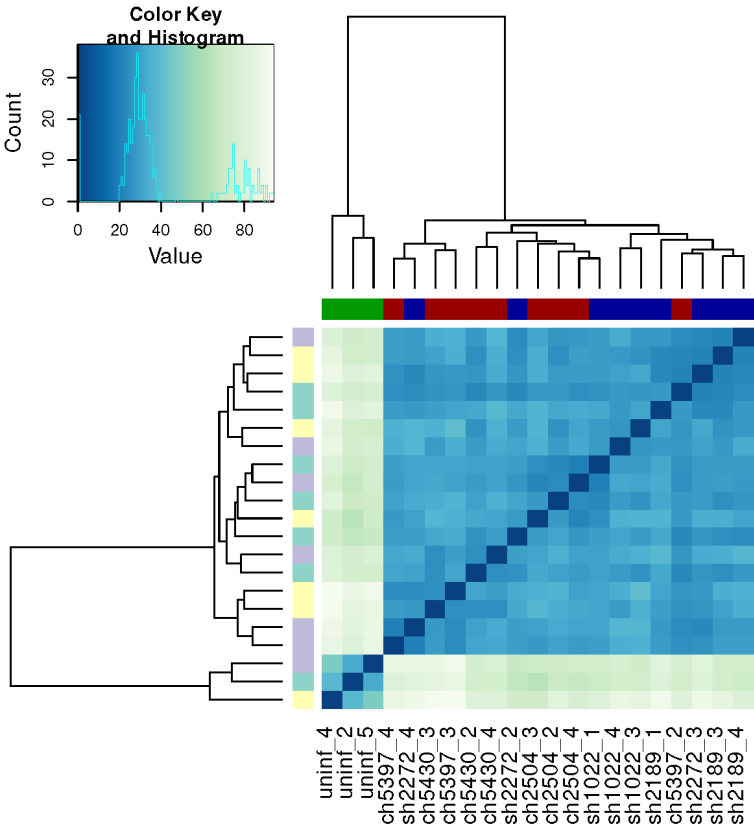

Supplement: Supplementary file 2 [file DataSheet_2.pdf]

Figure S2

A

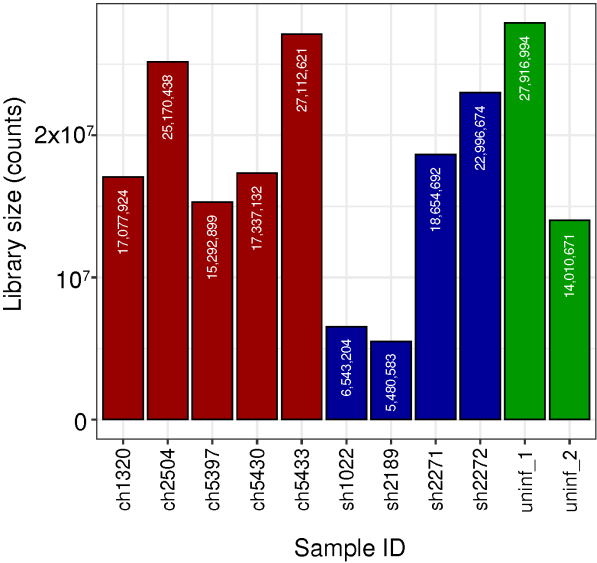

B

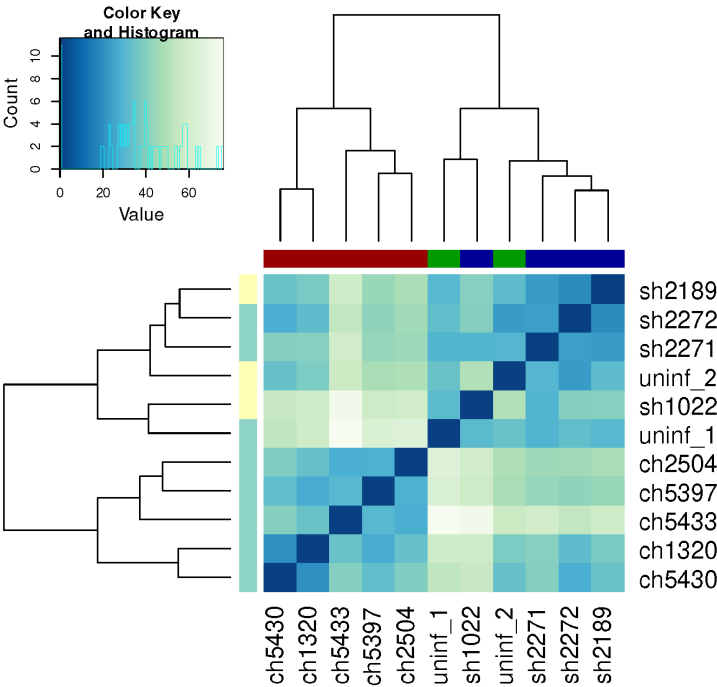

C

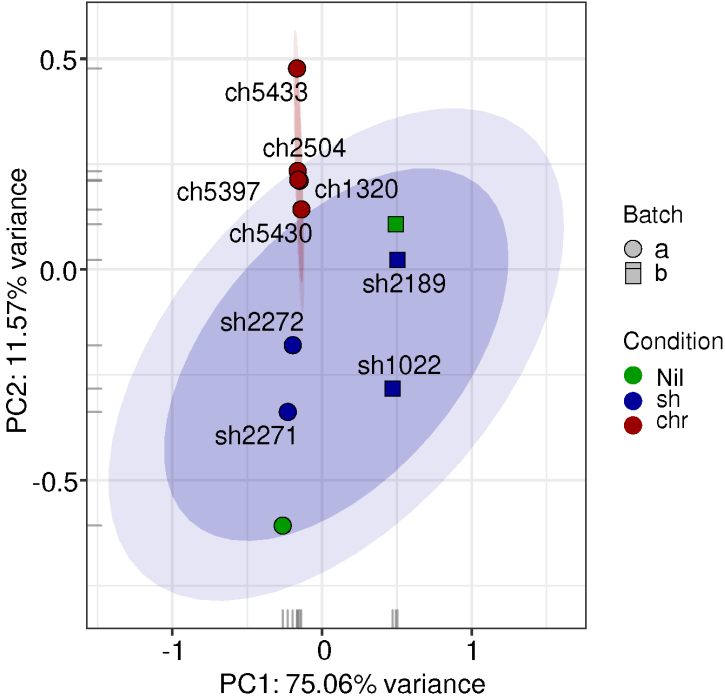

Supplement: Supplementary file 3 [file DataSheet_3.pdf]

Figure S3

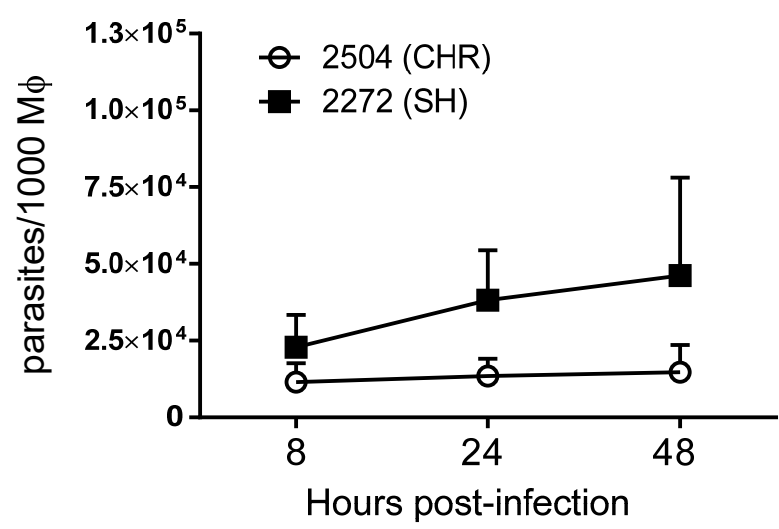

Supplement: Supplementary file 4 [file DataSheet_4.pdf]
