# Supplementary material for: Early Leukocyte Responses in Ex-Vivo Models of Healing and Non-Healing Human Leishmania (Viannia) panamensis Infections
Source: Front Cell Infect Microbiol. 2021 Sep 7;11:687607. doi: 10.3389/fcimb.2021.687607 (PMC8453012; doi:10.3389/fcimb.2021.687607)
Supplement: Supplementary file 5 [file Table_1.docx]

Table S1. Gene expression of inflammatory mediators in PBMCs and WBCs

|  | **PBMCs** | | | | |  | **WBCs** | | | | |
| --- | --- | --- | --- | --- | --- | --- | --- | --- | --- | --- | --- |
| **Gene**  **Symbol** | **Fold Change**  **(Infected/Uninfected)** | | | **mean** | **SD^a^** |  | **Fold Change (Infected/Uninfected)** | | | **mean** | **SD** |
|  | **Donor 1** | **Donor 2** | **Donor 3** |  |  |  | **Donor 1** | **Donor 2** | **Donor 3** |  |  |
| BCL6 | 2.51 | 2.57 | 1.83 | 2.30 | 0.41 |  | 1.32 | 1.42 | 1.23 | 1.33 | 0.09 |
| C3 | 10.24 | 12.91 | 13.68 | 12.28 | 1.81 |  | 5.82 | 6.73 | 3.59 | 5.38 | 1.61 |
| C3AR1 | 1.61 | 2.97 | 2.13 | 2.24 | 0.69 |  | 1.17 | 1.78 | 2.50 | 1.82 | 0.67 |
| CCL11 | NA^b^ | NA | NA |  |  |  | NA | NA | NA |  |  |
| CCL13 | 10.67 | 32.90 | 100.71 | 48.09 | 46.90 |  | 3.84 | 5.13 | 13.78 | 7.58 | 5.40 |
| CCL16 | NA | NA | NA |  |  |  | NA | NA | NA |  |  |
| CCL17 | 8.85 | 1.78 | 2.84 | 4.49 | 3.82 |  | 2.79 | 1.52 | 0.80 | 1.70 | 1.01 |
| CCL19 | 7.14 | 8.28 | 6.25 | 7.22 | 1.02 |  | NA | NA | NA |  |  |
| CCL2 | 46.08 | 42.52 | 288.81 | 125.80 | 141.18 |  | 3.01 | 8.06 | 9.61 | 6.89 | 3.45 |
| CCL21 | NA | NA | NA |  |  |  | NA | NA | NA |  |  |
| CCL22 | 4.16 | 3.76 | 1.45 | 3.12 | 1.46 |  | 1.13 | 1.42 | 0.72 | 1.09 | 0.35 |
| CCL23 | 1.97 | 4.47 | 2.12 | 2.85 | 1.40 |  | 1.14 | 2.19 | 5.75 | 3.03 | 2.42 |
| CCL24 | 22.25 | 45.89 | 13.97 | 27.37 | 16.56 |  | 8.12 | 26.54 | 5.56 | 13.41 | 11.45 |
| CCL3 | 11.05 | 15.89 | 24.83 | 17.26 | 6.99 |  | 3.90 | 6.77 | 9.54 | 6.74 | 2.82 |
| CCL4 | 1.99 | 4.29 | 4.58 | 3.62 | 1.41 |  | 2.07 | 3.18 | 11.03 | 5.43 | 4.89 |
| CCL5 | 0.83 | 0.79 | 0.62 | 0.75 | 0.11 |  | 1.04 | 1.04 | 1.24 | 1.11 | 0.12 |
| CCL7 | 341.56 | 661.68 | 2375.47 | 1126.24 | 1093.65 |  | 19.19 | 186.11 | 167.03 | 124.11 | 91.37 |
| CCL8 | NA | NA | NA |  |  |  | NA | NA | NA |  |  |
| CCR1 | 2.21 | 3.23 | 7.80 | 4.41 | 2.98 |  | 1.25 | 1.62 | 5.71 | 2.86 | 2.48 |
| CCR2 | 0.10 | 0.10 | 0.03 | 0.08 | 0.04 |  | 0.17 | 0.09 | 0.09 | 0.12 | 0.05 |
| CCR3 | 0.88 | 0.73 | 0.35 | 0.65 | 0.27 |  | 0.63 | 0.72 | 0.46 | 0.60 | 0.14 |
| CCR4 | 0.77 | 1.02 | 0.47 | 0.75 | 0.28 |  | 0.71 | 0.86 | 0.83 | 0.80 | 0.08 |
| CCR7 | 1.10 | 1.82 | 0.90 | 1.27 | 0.48 |  | 0.99 | 1.04 | 0.98 | 1.00 | 0.03 |
| CD14 | 0.44 | 0.74 | 0.49 | 0.56 | 0.16 |  | 0.47 | 0.40 | 0.56 | 0.48 | 0.08 |
| CD40 | 2.65 | 4.89 | 5.08 | 4.21 | 1.35 |  | 1.63 | 3.10 | 2.74 | 2.49 | 0.77 |
| CD40LG | 0.52 | 0.39 | 0.27 | 0.39 | 0.12 |  | 0.84 | 0.72 | 0.57 | 0.71 | 0.14 |
| CEBPB | 3.11 | 3.78 | 3.96 | 3.62 | 0.45 |  | 1.42 | 2.23 | 1.88 | 1.85 | 0.41 |
| CRP | NA | NA | NA |  |  |  | NA | NA | NA |  |  |
| CSF1 | 13.70 | 11.71 | 9.74 | 11.72 | 1.98 |  | 6.24 | 6.02 | 5.63 | 5.97 | 0.31 |
| CXCL1 | 16.98 | 65.80 | 97.95 | 60.24 | 40.77 |  | 1.32 | 8.11 | 1.76 | 3.73 | 3.80 |
| CXCL10 | 3.52 | 5.74 | 31.00 | 13.42 | 15.26 |  | 1.83 | 2.40 | 16.61 | 6.95 | 8.37 |
| CXCL2 | 5.19 | 13.27 | 22.22 | 13.56 | 8.52 |  | 2.00 | 4.00 | 5.59 | 3.87 | 1.80 |
| CXCL3 | 6.80 | 17.75 | 27.74 | 17.43 | 10.47 |  | 2.24 | 4.23 | 11.66 | 6.04 | 4.97 |
| CXCL5 | 43.59 | 135.30 | 80.67 | 86.52 | 46.13 |  | 14.05 | 47.50 | 7.43 | 22.99 | 21.48 |
| CXCL6 | 0.84 | 5.43 | 1.47 | 2.58 | 2.48 |  | 0.43 | 1.31 | 0.22 | 0.65 | 0.58 |
| CXCL9 | 1.08 | 2.77 | 5.33 | 3.06 | 2.14 |  | 1.46 | 2.00 | 3.62 | 2.36 | 1.12 |
| CXCR1 | 0.23 | 0.70 | 0.21 | 0.38 | 0.28 |  | 0.28 | 0.24 | 0.12 | 0.22 | 0.08 |
| CXCR2 | 0.39 | 0.47 | 0.27 | 0.38 | 0.10 |  | 0.28 | 0.43 | 0.10 | 0.27 | 0.16 |
| CXCR4 | 1.13 | 0.98 | 0.54 | 0.88 | 0.31 |  | 0.90 | 0.87 | 1.07 | 0.95 | 0.11 |
| FASLG | 1.06 | 1.13 | 1.07 | 1.09 | 0.03 |  | 1.10 | 1.09 | 0.85 | 1.01 | 0.14 |
| FOS | 0.39 | 0.31 | 0.45 | 0.38 | 0.07 |  | 0.37 | 0.28 | 0.21 | 0.29 | 0.08 |
| IFNG | 0.78 | 1.79 | 2.32 | 1.63 | 0.78 |  | 1.67 | 3.23 | 1.00 | 1.96 | 1.14 |
| IL10 | 0.92 | 1.23 | 2.72 | 1.63 | 0.96 |  | 0.40 | 0.52 | 1.17 | 0.70 | 0.41 |
| IL10RB | 1.19 | 1.48 | 1.61 | 1.43 | 0.21 |  | 1.17 | 1.13 | 1.18 | 1.16 | 0.02 |
| IL15 | 0.87 | 1.64 | 1.87 | 1.46 | 0.52 |  | 1.18 | 1.45 | 1.58 | 1.41 | 0.21 |
| IL17A | NA | NA | NA |  |  |  | NA | NA | NA |  |  |
| IL18 | 2.34 | 2.28 | 2.86 | 2.49 | 0.32 |  | 1.67 | 1.80 | 1.03 | 1.50 | 0.41 |
| IL1A | 9.75 | 11.79 | 7.33 | 9.63 | 2.23 |  | 1.93 | 9.25 | 3.13 | 4.77 | 3.93 |
| IL1B | 11.28 | 17.75 | 22.07 | 17.04 | 5.43 |  | 1.22 | 9.38 | 1.82 | 4.14 | 4.55 |
| IL1R1 | 0.91 | 2.46 | 1.06 | 1.48 | 0.86 |  | 1.79 | 1.74 | 4.51 | 2.68 | 1.59 |
| IL1RAP | 1.24 | 0.77 | 0.55 | 0.85 | 0.35 |  | 0.78 | 0.60 | 0.60 | 0.66 | 0.10 |
| IL1RN | 35.41 | 35.51 | 142.42 | 71.11 | 61.75 |  | 14.24 | 18.00 | 46.98 | 26.41 | 17.92 |
| IL22 | NA | NA | 1.15 |  |  |  | NA | NA | NA |  |  |
| IL23A | 0.49 | 0.57 | 0.56 | 0.54 | 0.05 |  | 0.97 | 0.75 | 1.11 | 0.94 | 0.18 |
| IL23R | NA | NA | NA |  |  |  | NA | NA | NA |  |  |
| IL5 | 0.53 | 0.68 | 0.83 | 0.68 | 0.15 |  | 1.00 | 1.04 | 0.51 | 0.85 | 0.30 |
| IL6 | 2.71 | 37.79 | 32.54 | 24.34 | 18.92 |  | 1.56 | 8.94 | 1.70 | 4.07 | 4.22 |
| IL6R | 0.83 | 1.27 | 1.03 | 1.04 | 0.22 |  | 0.85 | 1.07 | 1.00 | 0.97 | 0.11 |
| IL8 | 15.52 | 18.13 | 52.13 | 28.59 | 20.43 |  | 2.50 | 5.54 | 2.96 | 3.67 | 1.64 |
| IL9 | NA | NA | NA |  |  |  | NA | NA | NA |  |  |
| ITGB2 | 0.77 | 0.63 | 0.47 | 0.63 | 0.15 |  | 0.95 | 0.69 | 0.72 | 0.79 | 0.15 |
| KNG1 | NA | NA | NA |  |  |  | NA | NA | NA |  |  |
| LTA | 0.79 | 1.30 | 1.44 | 1.18 | 0.34 |  | 1.05 | 1.43 | 0.61 | 1.03 | 0.41 |
| LTB | 1.24 | 0.73 | 0.63 | 0.87 | 0.33 |  | 1.05 | 1.21 | 0.90 | 1.06 | 0.16 |
| LY96 | 1.30 | 1.06 | 1.43 | 1.26 | 0.18 |  | 1.20 | 0.99 | 1.64 | 1.28 | 0.33 |
| MYD88 | 1.51 | 1.89 | 2.42 | 1.94 | 0.46 |  | 1.63 | 1.67 | 2.23 | 1.84 | 0.33 |
| NFKB1 | 1.48 | 2.03 | 1.52 | 1.68 | 0.31 |  | 1.30 | 1.59 | 1.25 | 1.38 | 0.18 |
| NOS2 | NA | NA | NA |  |  |  | NA | NA | NA |  |  |
| NR3C1 | 1.28 | 1.25 | 0.83 | 1.12 | 0.25 |  | 1.29 | 1.25 | 1.22 | 1.25 | 0.03 |
| PTGS2 | 0.36 | 0.68 | 0.96 | 0.67 | 0.30 |  | 0.41 | 0.48 | 0.24 | 0.38 | 0.12 |
| RIPK2 | 2.39 | 3.39 | 4.67 | 3.48 | 1.14 |  | 1.79 | 2.41 | 6.84 | 3.68 | 2.75 |
| SELE | NA | NA | NA |  |  |  | NA | NA | NA |  |  |
| TIRAP | 0.61 | 0.89 | 0.53 | 0.67 | 0.19 |  | 0.71 | 0.68 | 0.41 | 0.60 | 0.16 |
| TLR1 | 0.77 | 0.97 | 1.14 | 0.96 | 0.18 |  | 0.68 | 0.93 | 0.38 | 0.66 | 0.28 |
| TLR2 | 1.04 | 1.62 | 1.03 | 1.23 | 0.34 |  | 1.25 | 0.93 | 1.32 | 1.17 | 0.21 |
| TLR3 | 0.79 | 1.30 | 0.97 | 1.02 | 0.26 |  | 1.02 | 0.54 | 1.41 | 0.99 | 0.44 |
| TLR4 | 0.73 | 1.05 | 1.39 | 1.05 | 0.33 |  | 0.74 | 0.68 | 1.14 | 0.86 | 0.25 |
| TLR5 | 0.38 | 0.50 | 0.50 | 0.46 | 0.07 |  | 0.52 | 0.43 | 0.71 | 0.56 | 0.15 |
| TLR6 | 0.43 | 0.64 | 0.54 | 0.54 | 0.11 |  | 0.66 | 0.56 | 0.36 | 0.52 | 0.15 |
| TLR7 | 0.37 | 0.41 | 0.65 | 0.48 | 0.15 |  | 0.20 | 0.15 | 0.21 | 0.19 | 0.03 |
| TLR9 | 0.87 | 0.69 | 0.24 | 0.60 | 0.33 |  | 0.39 | 1.41 | 0.34 | 0.71 | 0.61 |
| TNF | 1.62 | 1.87 | 3.33 | 2.27 | 0.92 |  | 0.89 | 1.53 | 1.46 | 1.29 | 0.35 |
| TNFSF14 | 2.60 | 3.73 | 2.01 | 2.78 | 0.88 |  | 2.06 | 4.14 | 3.08 | 3.09 | 1.04 |
| TOLLIP | 1.08 | 1.20 | 1.07 | 1.12 | 0.07 |  | 1.02 | 1.06 | 1.22 | 1.10 | 0.11 |

1. SD: Standard deviation
2. NA: Not amplified
